# A Skin Cancer Prevention Facial-Aging Mobile App for Secondary Schools in Brazil: Appearance-Focused Interventional Study

**DOI:** 10.2196/mhealth.9794

**Published:** 2018-03-09

**Authors:** Titus Josef Brinker, Marlene Heckl, Martina Gatzka, Markus V Heppt, Henrique Resende Rodrigues, Sven Schneider, Wiebke Sondermann, Carolina de Almeida e Silva, Michael C Kirchberger, Joachim Klode, Alexander H Enk, Sarah Knispel, Christof von Kalle, Ingo Stoffels, Dirk Schadendorf, Yasuhiro Nakamura, Stefan Esser, Aisllan Assis, Breno Bernardes-Souza

**Affiliations:** ^1^ Department of Dermatology University Hospital Heidelberg University of Heidelberg Heidelberg Germany; ^2^ Department of Translational Oncology National Center for Tumor Diseases German Cancer Research Center Heidelberg Germany; ^3^ Department of Dermatology University Medical Center Munich University of Munich Munich Germany; ^4^ Department of Dermatology and Allergic Diseases Ulm University Hospital University of Ulm Ulm Germany; ^5^ School of Medicine Federal University of Ouro Preto Ouro Preto Brazil; ^6^ Mannheim Institute of Public Health Social and Preventive Medicine, Medical Faculty Mannheim Heidelberg University Mannheim Germany; ^7^ Department of Dermatology, Venereology and Allergology University-Hospital Essen University of Duisburg-Essen Essen Germany; ^8^ Department of Dermatology University Hospital Erlangen Friedrich-Alexander-University Erlangen-Nürnberg Erlangen Germany; ^9^ Department of Skin Oncology/Dermatology Saitama Medical University International Medical Center Saitama Germany

**Keywords:** skin neoplasms, primary prevention, adolescent, schools, students, medical, mobile applications, skin aging, smartphone

## Abstract

**Background:**

The incidence of melanoma is increasing faster than any other major cancer both in Brazil and worldwide. Southeast Brazil has especially high incidences of melanoma, and early detection is low. Exposure to ultraviolet (UV) radiation is a primary risk factor for developing melanoma. Increasing attractiveness is a major motivation among adolescents for tanning. A medical student-delivered intervention that takes advantage of the broad availability of mobile phones and adolescents’ interest in their appearance indicated effectiveness in a recent study from Germany. However, the effect in a high-UV index country with a high melanoma prevalence and the capability of medical students to implement such an intervention remain unknown.

**Objective:**

In this pilot study, our objective was to investigate the preliminary success and implementability of a photoaging intervention to prevent skin cancer in Brazilian adolescents.

**Methods:**

We implemented a free photoaging mobile phone app (Sunface) in 15 secondary school classes in southeast Brazil. Medical students “mirrored” the pupils’ altered 3-dimensional (3D) selfies reacting to touch on tablets via a projector in front of their whole grade accompanied by a brief discussion of means of UV protection. An anonymous questionnaire capturing sociodemographic data and risk factors for melanoma measured the perceptions of the intervention on 5-point Likert scales among 356 pupils of both sexes (13-19 years old; median age 16 years) in grades 8 to 12 of 2 secondary schools in Brazil.

**Results:**

We measured more than 90% agreement in both items that measured motivation to reduce UV exposure and only 5.6% disagreement: 322 (90.5%) agreed or strongly agreed that their 3D selfie motivated them to avoid using a tanning bed, and 321 (90.2%) that it motivated them to improve their sun protection; 20 pupils (5.6%) disagreed with both items. The perceived effect on motivation was higher in female pupils in both tanning bed avoidance (n=198, 92.6% agreement in females vs n=123, 87.2% agreement in males) and increased use of sun protection (n=197, 92.1% agreement in females vs n=123, 87.2% agreement in males) and independent of age or skin type. All medical students involved filled in a process evaluation revealing that they all perceived the intervention as effective and unproblematic, and that all pupils tried the app in their presence.

**Conclusions:**

The photoaging intervention was effective in changing behavioral predictors for UV protection in Brazilian adolescents. The predictors measured indicated an even higher prospective effectiveness in southeast Brazil than in Germany (>90% agreement in Brazil vs >60% agreement in Germany to both items that measured motivation to reduce UV exposure) in accordance with the theory of planned behavior. Medical students are capable of complete implementation. A randomized controlled trial measuring prospective effects in Brazil is planned as a result of this study.

## Introduction

According to the World Health Organization, the incidence of melanoma is increasing more rapidly than any other major cancer both in Brazil and worldwide. Melanoma is one of the most common cancers in young adults and poses substantial health and economic burdens [1].

Approximately 90% of melanomas are associated with ultraviolet (UV) exposure, in particular with the frequency of severe sunburns, and are therefore eminently preventable [2]. Multiple studies showed that daily use of a sunscreen with a sun protection factor above 30, as recommended by international dermatology guidelines, may prevent sunburns and skin cancer, including melanoma [3-6].

Brazil has one of the highest UV indexes on earth; additionally, tanning is culturally established, and Brazilians commonly experience unprotected overexposure to the sun, especially in their childhood and teenage years [7-11]. In a 2008 population-based survey with 1604 participants in the south of Brazil, 48.7% reported at least one sunburn in the prior year [10]. In an attempt to mitigate the health damage caused by excessive UV exposure, Brazil was the first country to prohibit indoor tanning in 2009, albeit with limited success [9]. The southeast of Brazil (the location of this study) is especially populated by citizens with a European ancestry and therefore has high incidences of melanoma (up to 23.5/100,000 inhabitants) with a lack of early diagnosis and an overall survival rate below worldwide rates [12-15].

Interventions encouraging sun protection habits are important, particularly among adolescents, as increased risk of skin cancer is associated with cumulative UV exposure and sunburns early in life [16-18]. In line with this association, recent experimental studies to test these effects in young target groups aimed at promoting sunscreen use as an end point [19-22], and others used various UV protection behaviors (including avoiding sunbeds) or behavior scores [23-34]. Given the substantial amount of time that children and adolescents of all social backgrounds spend in the school environment, addressing skin cancer prevention in this setting is crucial and provides a unique opportunity to propel skin cancer prevention programs [35].

### Current Knowledge on School-Based Skin Cancer Prevention

Unhealthy behavior with respect to UV exposure is mostly initiated in early adolescence [36], commonly with the belief that a tan increases attractiveness [26,37,38], and the problems related to melanoma and skin atrophy are too far in the future for them to fathom.

A recent randomized trial with Australian high school students demonstrated that appearance-based videos on UV-induced premature aging were superior in encouraging sunscreen use to videos of the same length focusing exclusively on health aspects [19]. These findings are in line with international studies demonstrating the important influence of self-perceived attractiveness on self-esteem in adolescence [39,40]. Furthermore, enhancing one’s attractiveness is a primary motivation for tanning in adolescents both in Brazil and worldwide [36,37,41]. In addition, the success of appearance-based photoaging intervention mobile apps, in which an image is altered to predict future appearance, in the fields of tobacco and adiposity prevention have shown promise for these interventions in behavioral change settings [42-47].

In the setting of melanoma prevention, a quasi-experimental study by Williams et al demonstrated significantly higher scores for predictors of sun protection behavior in young women from the United Kingdom (70 participants in total) using a photoaging desktop program [48]. Furthermore, the photoaging software showed a promising reduction in young adults’ tanning intentions in a study with 10 participants in total (7 female and 3 male) [49]. However, prior studies were limited by their small sample size and limitations related to expanding the target population.

### Introduction to the Sunface App

**Figure 1:**
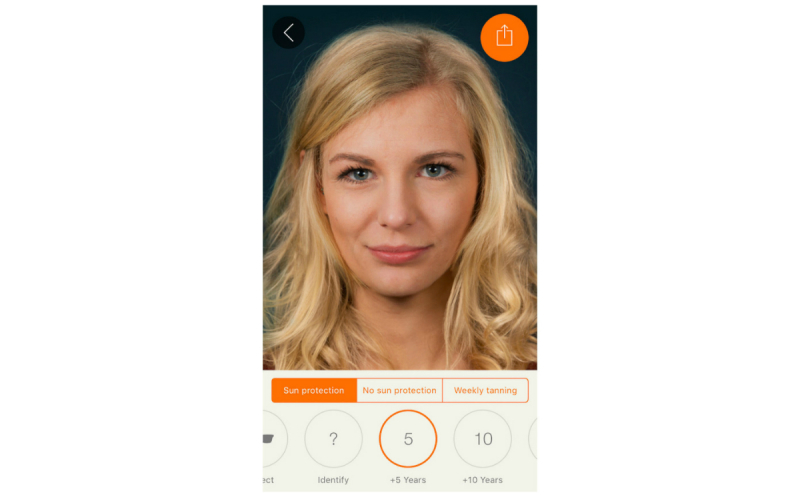
Effect view: 5 years of skin aging with sun protection.

**Figure 2:**
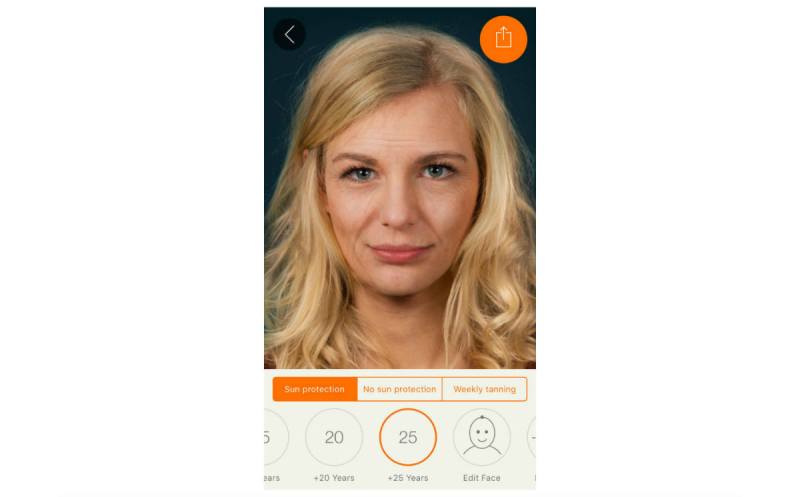
Effect view: 25 years of skin aging with sun protection.

**Figure 3:**
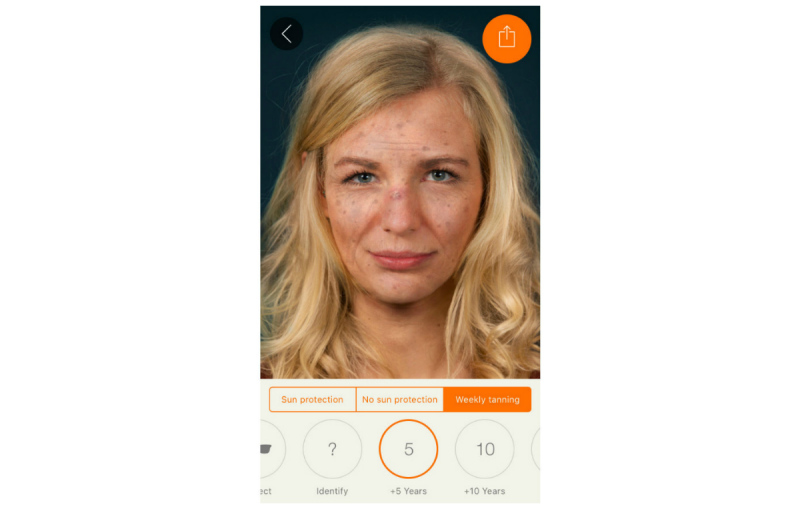
Effect view: 5 years of weekly tanning without sun protection.

**Figure 4:**
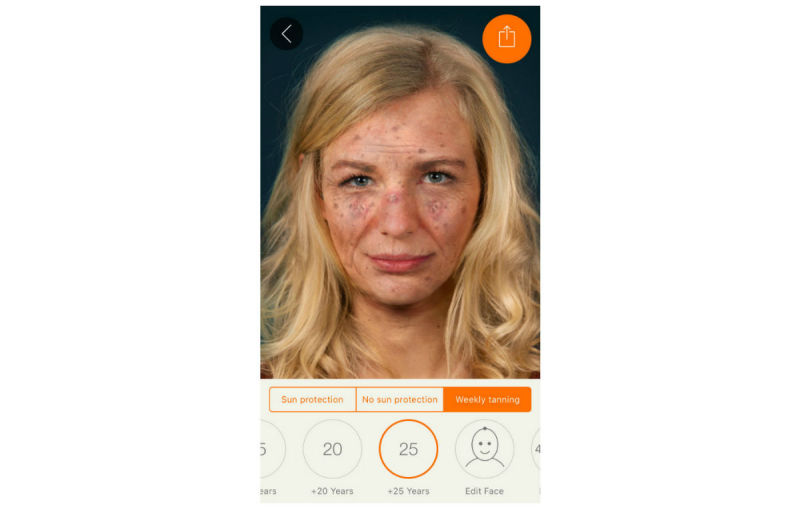
Maximum effect view: 25 years of UV damage due to weekly tanning.

**Figure 5:**
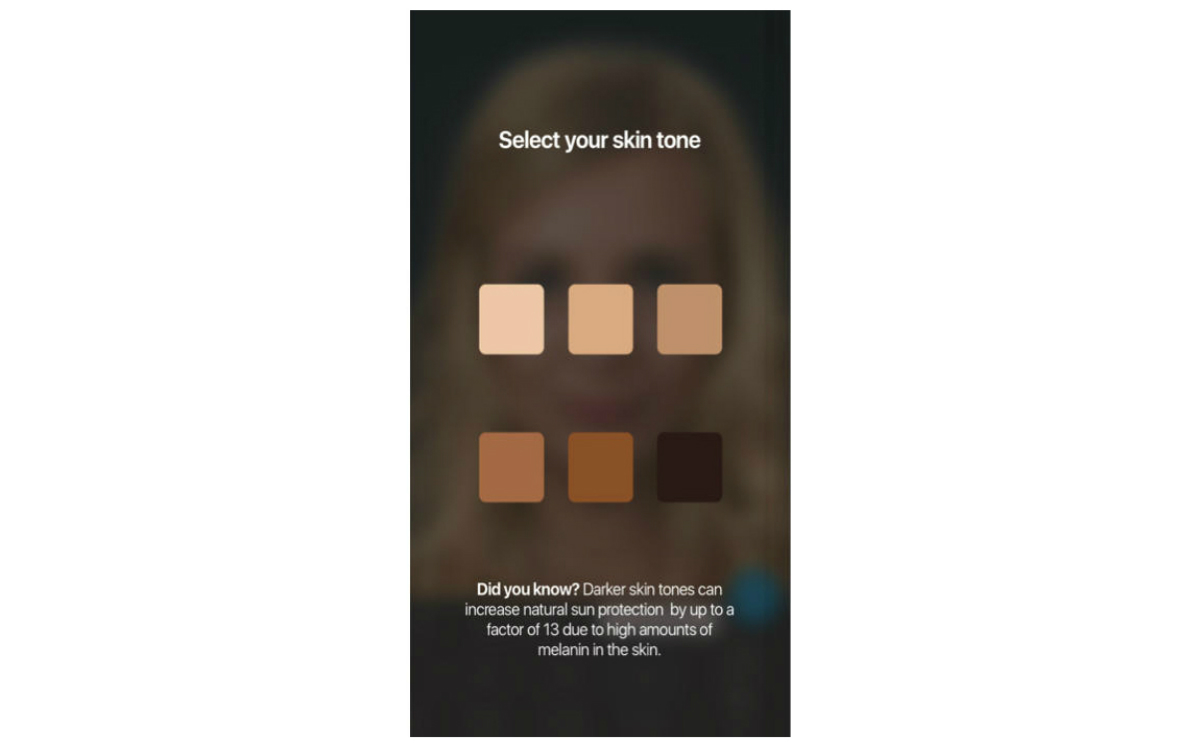
Start screen of the app prompts users to pick their skin type.

We harnessed the widespread availability of mobile phones and adolescents’ interest in appearance to develop the free mobile phone app Sunface, which enables the user to take a selfie and then offers a choice of 3 categories: daily sun protection, no sun protection, and weekly tanning, showing the altered face at 5 to 25 years in the future (Figures 1, 2, 3, and 4). All effects are based on the individual skin type that the user can choose at the start of the app (Figure 5). The app also shows the most common UV-induced skin cancers via extra buttons and calculates how the odds ratio is increased with different behaviors.

In addition, the app gives advice on sun protection, explains the facial changes, and encourages skin examinations using the ABCDE rule (asymmetry, border irregularity, color variety, diameter, and evolution [50]).

Afterward, the app offers many options for sharing (animated photo or video; see Multimedia Appendix 1) with family and friends. By this means, the social network of the user may also be informed about the various photoaging effects of excessive UV exposure and potential health consequences, as well as potentially learning about the benefits of using the app [34].

**Figure 6:**
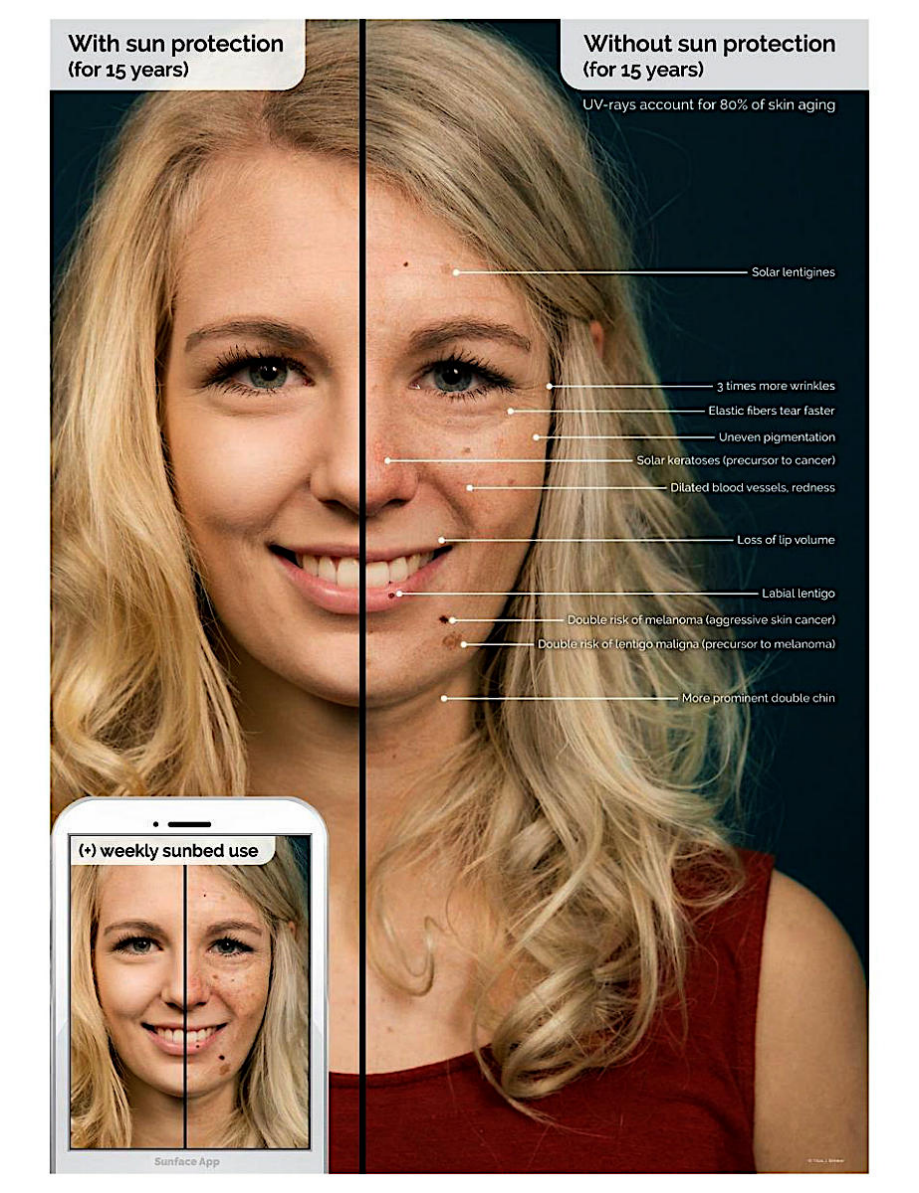
Explanatory graphic of the effects within the app.

To produce realistic effects (Figure 6) and to show the user realistic odds ratios for the options they choose in the app for the three most strongly associated skin pathologies, an extensive review of the literature on UV-induced skin damage [51,52] was conducted for each specific skin type. As no trials with 25 years of follow-up were available, we had to extrapolate the evidence on UV-induced skin damage for the specific skin types. The evidence consists of more than 50 publications to create realistic effects from a clinician’s standpoint (which may differ from what the average person perceives as realistic).

We recently implemented this app in 2 German secondary schools via a method called mirroring. Mirroring means that the student’s altered 3-dimensional (3D) selfies are “mirrored” via a projector in front of the entire class. Using an anonymous questionnaire, we then measured sociodemographic data and risk factors for melanoma, as well as the perceptions of the intervention on a 5-point Likert scale among 205 students of both sexes aged 13 to 19 years (median 15 years).

In our pilot study, we found more than 60% agreement in both items measuring motivation to reduce UV exposure and only 12.5% disagreement: 126 (63.0%) agreed or strongly agreed that their 3D selfie motivated them to avoid using a tanning bed, and 124 (61.7%) agreed or strongly agreed to increase their use of sun protection; only 25 (12.5%) disagreed with both items. [33].

An explanation for these results is offered by the theory of planned behavior, according to which the subjective norm (eg, “my friends think that tanning makes you unattractive”), attitudes (consisting of beliefs, such as “tanning leads to unattractiveness”), and perceived behavioral control (eg, “I can apply sunscreen correctly”) influence both the behavioral intentions of a person and his or her behavior. Photoaging interventions have the potential to affect all three of these predictors, and the mirroring intervention specifically had a strong influence on the subjective norm in the previous pilot study [33].

This study investigated whether the results of our novel photoaging intervention would be reproducible in Brazil, a country with a high UV index and, thus, higher prevalences of malignant melanoma and an even stronger need for effective skin cancer prevention programs. Also, a process evaluation investigated whether volunteering medical students would be capable of complete implementation.

## Methods

### Setting

We conducted the study in 2 regular public secondary schools in the city of Ponte Nova, southeast Brazil. Students who were 13 to 19 years of age and attending regular secondary schools in the city of Ponte Nova were eligible.

### Intervention

The mirroring approach was implemented by medical students from the Education Against Tobacco nonprofit organization who were attending the Federal University of Ouro Preto in Brazil [46,53]. To increase the pupils’ familiarity with the Sunface photoaging app and their participation in the mirroring intervention, we asked them to download the app before our visit, via a letter 1 week in advance. When we visited the schools, 12.6% (45/356) had the app on their mobile phones.

The intervention consisted of a 45-minute app-based mirroring educational module in the classroom setting. It was presented by 2 medical students per classroom to approximately 24 students at a time (mean 23.7, SD 6.1 students).

In the first 10-minute phase, the displayed face of one student volunteer was used to show the app’s altering features to the peer group, providing an incentive for the rest of the class to test the app. In front of their peers and teachers, students could interact with their own animated face via touch (coughing, sneezing, etc) and display their future self based on their skin type and use of sun protection or tanning beds 5, 10, 15, 20, or 25 years in the future. Multiple device displays could be projected simultaneously, which we used to consolidate the altering measures with graphics (eg, to explain skin atrophy and solar elastosis). We implemented mirroring with 10 Galaxy Tab A tablets (Samsung, Seoul, South Korea) via Apple’s AirPlay interface (Apple Inc) using the app Mirroring360 (Splashtop Inc) for the Android operating system (Google Inc).

In the second 15-minute phase, students were encouraged to try the app on one of the tablet computers. We calculated the number of provided tablet computers so that this phase would take up to 12 minutes at most after factoring in a use time of approximately 4 minutes per student. By this calculation, 25 minutes of the mirroring intervention and 10 provided tablets were sufficient to have every student within a class of 40 pupils successfully photoaged at least once.

In the following 15 minutes, the medical students discussed the remaining functions of the app with the students: facial changes, the ABCDE rule, and the guidelines for sun protection were addressed in an interactive setting. In the last 5 minutes, we measured the students’ perception of the intervention via an anonymous paper-and-pencil questionnaire.

### Data Collection

We measured the students’ sociodemographic data (sex, age, school type) and their risk profile (skin type, sex, age, sunburn in the past, sunbed use) directly after the intervention via an anonymous survey. The reactions to the intervention were captured via 6 items on 5-point Likert scales: (1) increase of UV protection intentions due to the photoaging intervention (2 items: indoor vs outdoor tanning); (2) perceived reactions of the peer group on change in attractiveness (2 items: indoor vs outdoor tanning), whether they perceived the intervention as fun (1 item), and the effects of the app as realistic (1 item).

The items used were transferred from previously published studies [33,43,54] and pretested in advance in accordance with the guidelines for good epidemiologic practice [55].

The medical students filled out a brief process evaluation consisting of 6 items capturing the complete implementation of the intervention, as well as how the medical students perceived its effectiveness when in class.

## Results

### Participants

We included 356 Brazilian secondary school students of both sexes in the age group of 13 to 19 years (mean 15.95, SD 1.73 years; 141/356, 39.7% male; 214/356, 60.3% female) in this cross-sectional pilot study. They were attending 2 regular public secondary schools in the city of Ponte Nova in southeast Brazil. Almost all participants (336/356, 94.4%) owned a smartphone.

From a risk profile standpoint, 43.9% (156/356) of the participants had a Fitzpatrick skin type of 1 or 2 [56]; indoor tanning bed use in the past year was reported by 2.0% (7/356) and use at least once in their life was reported by 4.5% (16/356) [57]. Most students (205/356, 57.6%) remembered at least one sunburn in the past [58], 18.3% (65/356) reported one or more sunburns in the last 12 months, and 15.8% (56/356) reported that they frequently went out in the sun to get a tan.

**Figure 7:**
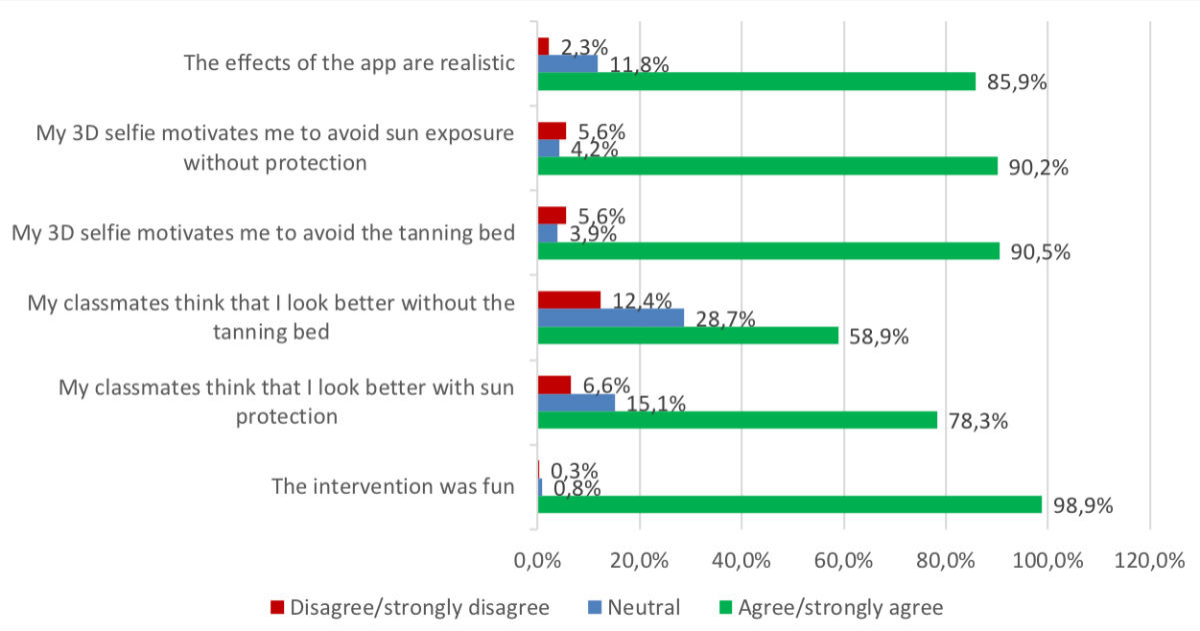
Overall results of the whole sample. 3D: 3-dimensional.

**Figure 8:**
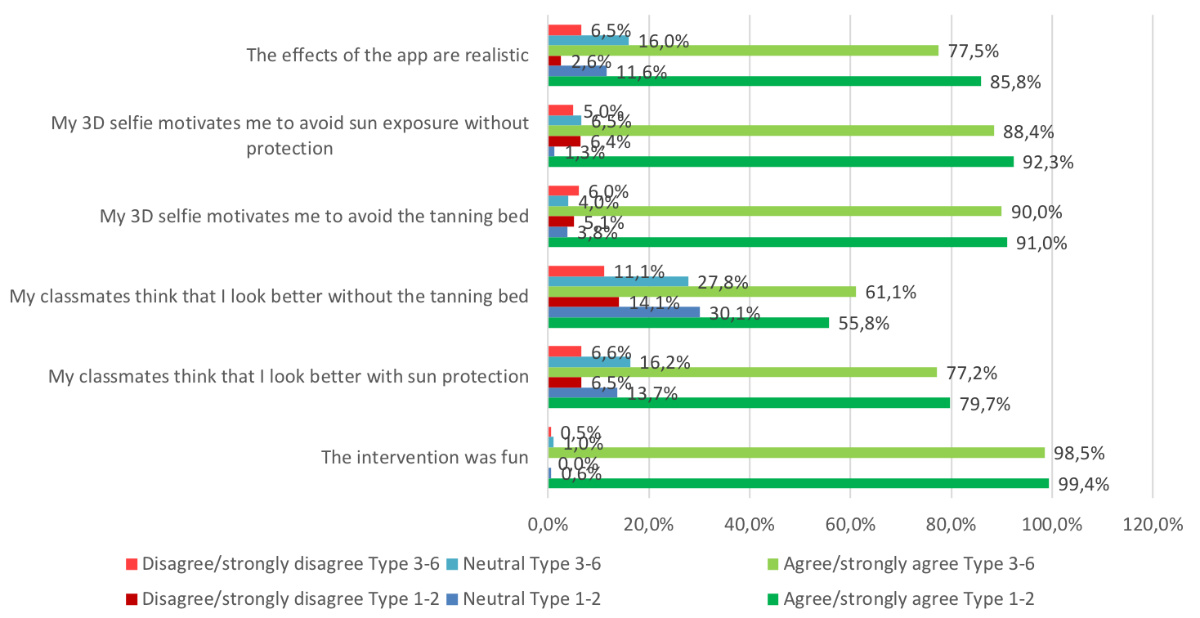
Results in Fitzpatrick skin types 1-2 versus 3-6. 3D: 3-dimensional.

**Figure 9:**
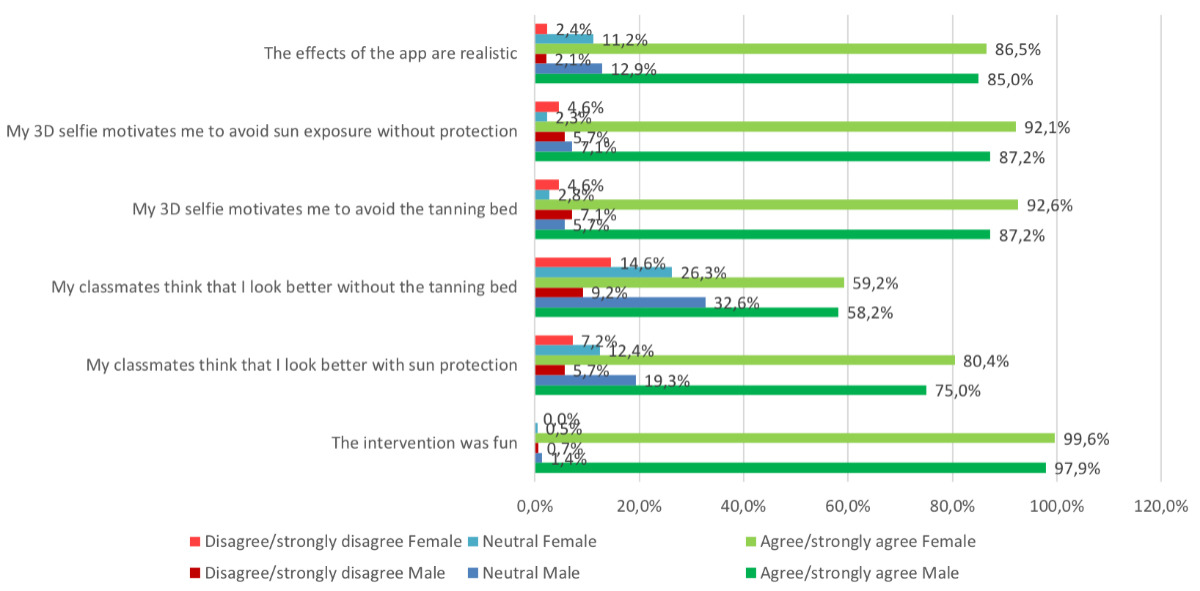
Results in male versus female participants. 3D: 3-dimensional.

**Figure 10:**
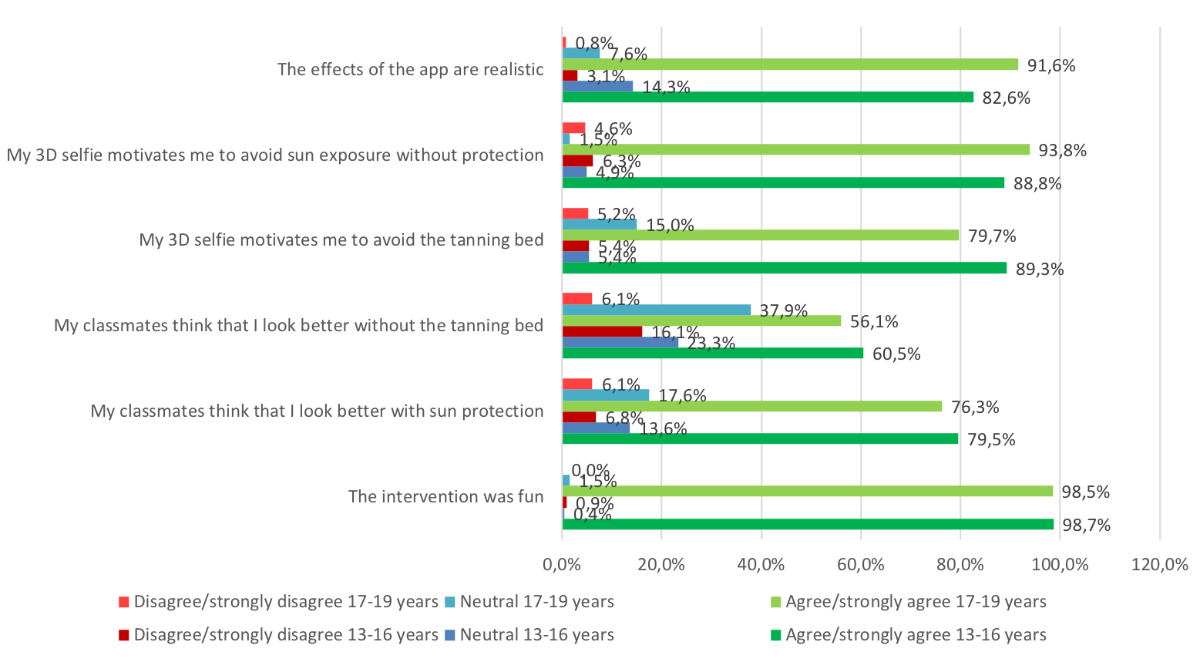
Results in 13- to 16-year-old versus 17- to 19-year-old participants. 3D: 3-dimensional.

We analyzed and illustrated all data in regard to overall perceptions of the intervention within the whole sample (Figure 7), but also to learn about how well the app was received by students of different Fitzpatrick skin types (Figure 8), sex (Figure 9), and age groups (Figure 10).

### Realism of the Created Selfies

In our sample, we measured overall agreement with the subjective realism of the created selfies (n=305, 85.9% strongly agreed or agreed on realism, while only n=8, 2.3% disagreed or strongly disagreed; Figure 7). These results did not vary notably between male (n=119, 85.0% agreement; n=3, 2.1% disagreement) and female participants (n=185, 86.5% agreement; n=5, 2.4% disagreement; Figure 9). However, the 13- to 16-year-olds (n=185, 82.6% agreement; n=7, 3.1% disagreement) and those with skin types 1 and 2 (n=133, 85.8% agreement; n=4, 2.6% disagreement) tended to perceive the selfies as less realistic, as opposed to 17- to 19-year-olds (n=120, 91.6% agreement; n=1, 0.8% disagreement) and participants with skin types 3 to 6 (n=155, 77.5% agreement; n=13, 6.5% disagreement; Figure 8 and Figure 10). The group that reported at least one sunburn in the last 12 months had a 92.3% (n=60) agreement and 1.5% (n=1) disagreement compared with 84.5% (n=245) agreement and 2.4% (n=7) disagreement among participants without sunburns in the past 12 months.

### Motivation to Reduce Ultraviolet Exposure

We measured more than 90% agreement in both items that measured motivation to reduce UV exposure and only 5.6% disagreement (n=322, 90.5% agreed or fully agreed that their 3D selfie would motivate them to avoid the tanning bed; n=321, 90.2% agreed or fully agreed that they would increase their use of sun protection); only 20 (5.6%) disagreed or fully disagreed with both items. The perceived effect on motivation was similar between participants with different Fitzpatrick skin types in both tanning bed avoidance (n=142, 91.0% agreement in skin types 1-2 vs n=179, 90.0% agreement in types 3-6) and increased use of sun protection (n=144, 92.3% agreement in skin types 1-2 vs n=176, 88.4% agreement in types 3-6; Figure 8), and also similar between the age groups (Figure 10). Comparing by sex, the perceived effect on motivation was higher in female pupils in both tanning bed avoidance (n=198, 92.6% agreement in female vs n=123, 87.2% agreement in male participants) and increased use of sun protection (n=197, 92.1% agreement in female vs n=123, 87.2% agreement in male participants).

### Perceived Subjective Norm During the Mirroring Intervention

The 2 items measuring the perceived reactions of the peer group toward the individual selfie showed positive peer pressure in regard to both use of sun protection (n=275, 78.3%) and tanning bed avoidance (n=209, 58.9%; Figure 7). The subjective norm on decreasing UV exposure in order to look more attractive was similarly perceived between the different age groups (Figure 10). However, female participants (n=169, 80.4% agreement; n=15, 7.2% disagreement) tended to feel a stronger urge to increase the use of sun protection due to the behavior of their classmates than did male participants (n=105, 75% agreement; n=8, 5.7% disagreement). Participants with Fitzpatrick skin types 1 and 2 (n=87, 55.8% agreement; n=22, 14.1% disagreement) tended to perceive less peer pressure for avoiding tanning beds than did skin types 3 to 6 (n=121, 61.1% agreement; n=22, 11.1% disagreement). Participants with at least one sunburn in the last 12 months had a higher agreement in the increased use of sun protection item (n=49, 75.4% agreement; n=5, 7.7% disagreement vs n=197, 67.9% agreement; n=26, 9% disagreement in participants without sunburn in the past 12 months) and in the avoidance of sunbeds item (n=55, 84.6% agreement; n=5, 7.7% disagreement vs n=220, 76.9% agreement; n=18, 6.2% disagreement, respectively).

### Global Feedback

Most participants claimed that they perceived the intervention as fun (n=351, 98.9% agreement vs n=1, 0.3% disagreement), and this fraction of agreement was similar throughout all subgroups. Most participants (n=271, 77.0%) reported that they would try the app again later on, 283 (80.2%) planned to show the app to another person after school, and 352 (98.9%) agreed that they had learned new things about the advantages of sun protection.

### Data Obtained From the Medical Students

Our process evaluation conducted among all of the 6 volunteering medical students via a short questionnaire after every classroom visit revealed that 100% of the secondary school students received the mirroring intervention as outlined in the methods section, and that 100% of the medical students were capable of having an empathic communication with the students and regarded the intervention as enjoyable.

## Discussion

### Principal Findings

Our data showed that the mirroring intervention was effective in changing the predictors of behavior in young risk groups living in Brazil, a country with a high UV index and where tanning is culturally established. The predictors measured indicated an even higher prospective effectiveness in southeast Brazil than in Germany (>90% agreement in Brazil vs >60% agreement in Germany to both items that measured motivation to reduce UV exposure [33]).

While teledermatology [59,60] and, more specifically, skin cancer diagnostic apps [61-65] are emerging, early diagnostics may only be successful if a patient is sensitized for an eventual skin cancer risk and about skin cancer in general. Photoaging smartphone apps seem capable of filling this important gap by appealing to vanity.

### Interpretation

Available data on appearance-based behavioral change settings for adolescents reveal that photoaging interventions appear to be more effective for girls [46]. Also, data from our recent study in Germany indicated that the intervention was more effective in changing motivational predictors in those with Fitzpatrick skin types 1 and 2, as well as in older adolescents. In our sample, the perceived effect on motivation was higher among female pupils in both tanning bed avoidance (n=198, 92.6% agreement in female vs n=123, 87.2% agreement in male participants) and increased use of sun protection (n=197, 92.1% agreement in female vs n=123, 87.2% agreement in male participants), while it was independent of age or skin type. We hypothesize that the most likely explanation for this is that gender roles are more established in southeast Brazil than they are in Germany and that peer pressure plays a larger role, which thus flattened out the differences for age and skin type that we found in our German study [33]. Accordingly, this hypothesis is in line with the finding that an intervention like the mirroring intervention, which aims at yielding peer pressure effects and addresses social norms, had a larger impact in a country like Brazil, where social norms play a larger role than in Germany.

### Limitations

As we conducted this study only in Brazil, our results might not be generalizable to other cultural or national settings. However, cosmetics are used by adolescents in most countries and appearance is a strong motivator for behavior in different cultural contexts [39,66].

In addition, our results stemmed from anonymous self-reports via paper-and-pencil questionnaires filled out after the intervention. While anonymity decreases social desirability bias in self-reports, they may not be regarded to be as objective as externally measurable markers, such as biochemical findings or clinical observation. Furthermore, handing the questionnaires out after the intervention rather than before might provoke a social desirability bias despite anonymity.

### Conclusions

The photoaging intervention was effective in generating an increased intention for UV protective behavior in Brazilian adolescents. The predictors measured indicated an even higher prospective effectiveness in southeast Brazil than in Germany (>90% agreement in Brazil vs >60% agreement in Germany to both items that measured motivation to reduce UV exposure) in accordance with the theory of planned behavior. Medical students are capable of complete implementation. A randomized controlled trial measuring prospective effects in Brazil is planned as a result of this study [67].

## Appendix

Multimedia Appendix 1 Animated version of the Sunface App effect view.

## Abbreviations

3D: 3-dimensional
ABCDE: asymmetry, border irregularity, color variety, diameter, and evolution
UV: ultraviolet
